# Extended Duration of Transgene Expression from Pegylated POD Nanoparticles Enables Attenuation of Photoreceptor Degeneration 

**DOI:** 10.1371/journal.pone.0082295

**Published:** 2013-11-22

**Authors:** Christina Binder, Siobhan M. Cashman, Rajendra Kumar-Singh

**Affiliations:** Department of Ophthalmology, Tufts University School of Medicine, Boston, Massachusetts, United States of America; National Eye Institute, United States of America

## Abstract

Retinitis pigmentosa (RP) is the most genetically heterogeneous disorder known to cause blindness, involving over 50 different genes. Previously, we have described nanoparticles (NPs) 150 nm in size, comprised of a 3.5 kD peptide (POD) complexed to PEG and DNA (PEGPOD DNA). These NPs expressing GDNF enabled rescue of photoreceptor degeneration in mice up to 11 days post injection. In the current study we examine use of scaffold/ matrix attachment regions (S/MARs), CpG depletion and titration of DNA content of PEGPOD DNA NPs to extend the duration of transgene expression. S/MARs and CpGs did not significantly influence the duration of transgene expression, but did influence its stability. These parameters enabled us to extend transgene expression from 48 hours to 10 weeks. At 77 days post injection, we observed a 76% rescue of the thickness of the retinal outer nuclear layer (ONL) and at 37 days post injection we observed 53% and 55% rescue of the A and B wave ERG amplitudes respectively and 60% rescue of the ONL. Our studies suggest that PEGPOD DNA NPs have potential as gene delivery vectors for the retina.

## Introduction

Retinitis Pigmentosa (RP) is one of the most common causes of blindness among the working population, affecting approximately 1 in 4000 individuals [1]. RP is an extremely heterogeneous disorder, involving over 50 different genes with up to 680 mutations in a single gene (reviewed in 2). Although the mechanism of photoreceptor degeneration varies depending on the gene involved, the final stages of photoreceptor cell death in RP involve the activation of apoptosis [3]. There is currently no treatment available for RP. However, recent evidence from multiple ocular gene therapy clinical trials strongly suggests that gene transfer is likely to be highly beneficial to RP patients [4–8].

Currently, the most commonly used vectors for gene transfer to humans involve the use of recombinant viruses such as adenovirus (Ad), adeno-associated virus (AAV), lentivirus or retrovirus. While highly successful, there are some limitations in the use of recombinant viruses for gene therapy. For example, viral proteins are known to be highly immunogenic and limit levels of gene transfer by stimulation of an innate as well as an adaptive immune response [9]. Although the retinal pigment epithelium provides significant protection to the retina from inflammation [10], an immune response (albeit attenuated) to viral proteins is observed following subretinal delivery of viruses such as AAV and Ad [11]. Recombinant viruses have a relatively limited transgene cargo capacity. For example, AAV, the most commonly used vector in ocular gene therapy has a cargo capacity of approximately 4.8Kb [12]. While this capacity is sufficient to accommodate most cDNAs, it is insufficient to also accommodate large upstream regulatory regions deemed to be potentially necessary for optimal transgene expression. For example, haplo-insufficiency [13] or above normal levels of expression [14] of rhodopsin cause photoreceptor degeneration. Over-expression of ciliary neurotrophic factor (CNTF) from viruses has been found to be toxic to the retina [15]. Furthermore, the ratio of total viral protein per transgene delivered into the cell is unfavorable when considering viruses. These proteins are degraded into short peptides and displayed on the cell surface for immune surveillance. Production methods for generation of pure populations of recombinant viruses are not as robust as for peptides or small molecule drugs [16]. Despite these limitations, recombinant viruses are currently the vector of choice due to their advantages of robust infection rates and longevity of expression and a simple lack of alternative vectors. As is the case for recombinant viruses, non-viral vectors also have significant limitations. Chief amongst these is that non-viral vectors lack the ability to deliver genes efficiently to post mitotic cells such as photoreceptors or retinal pigment epithelium (RPE) - two cell types commonly involved in inherited ocular disorders such as RP and age-related macular degeneration. Indeed, there is currently very limited evidence of efficient non-viral gene transfer to post-mitotic photoreceptors (discussed in detail later). Secondly, the longevity of gene expression from non-viral vectors is generally very limited relative to recombinant viruses [16]. 

Viruses are efficient gene transfer vectors because they have evolved specific mechanisms to overcome the barriers of the cell membrane, endosomal escape, nuclear targeting as well as stabilizing of their genome inside the nucleus [9]. A study of these mechanisms has provided clues for the development of non-viral vectors that may also overcome the above barriers. Recently, we have described a 3.5Kd peptide referred to as POD (Peptide for Ocular Delivery) whose design is based on the glycosaminoglycan interactions of acidic and basic fibroblast growth factor, two proteins abundantly present in ocular tissues. POD is a positively charged peptide that can enter cells without disrupting the cell membrane [17] and is capable of escaping the endosome and entering the nucleus [17]. We have demonstrated that when POD is chemically conjugated to small molecules, POD can deliver those molecules to the RPE, photoreceptors, ganglion cells, corneal epithelial cells etc. *in vivo* [17]. When recombinant proteins such as green fluorescent protein (GFP) are synthesized as fusion proteins containing the POD sequence, those recombinant proteins can also enter photoreceptors, RPE, ganglion cells, corneal epithelial cells etc. *in vivo* [18]. Finally, we have shown that POD can deliver siRNAs or plasmid DNA *in vitro* without the need for chemical conjugation [17,19]. For delivery of plasmid DNA *in vivo*, we developed an alternative approach involving the pegylation of POD with a 10 Kd polyethylene glycol (PEG) to form homogeneous PEGPOD DNA nanoparticles (NPs) of approximately 150 nm in size, not substantially larger than an adenovirus capsid of approximately 100nm [19]. Such PEGPOD DNA NPs were utilized to deliver a plasmid expressing various marker genes or glial cell line-derived neurotrophic factor (GDNF) to Balb/C mice undergoing blue light induced photoreceptor apoptosis [20]. These were among the first studies demonstrating non-viral gene transfer as being efficacious in attenuating photoreceptor apoptosis and subsequent degeneration [20]. However, longevity of gene expression was limited and enabled a rescue of the photoreceptors and the scotopic ERG only when the NPs were injected within 4 days of blue light exposure. The motivation for the current studies was to extend the duration of transgene expression from PEGPOD DNA NPs from 48 hours to several months. While not sufficient to justify a move towards clinical trials or to be deemed competitive with viruses, such extension of duration of transgene expression would be an important step forward towards the development of PEGPOD DNA NPs as non-viral gene transfer vectors for retinal degeneration.

## Results

### Duration of transgene expression from PEGPOD DNA nanoparticles is inversely correlated with DNA concentration

**Figure 1:**
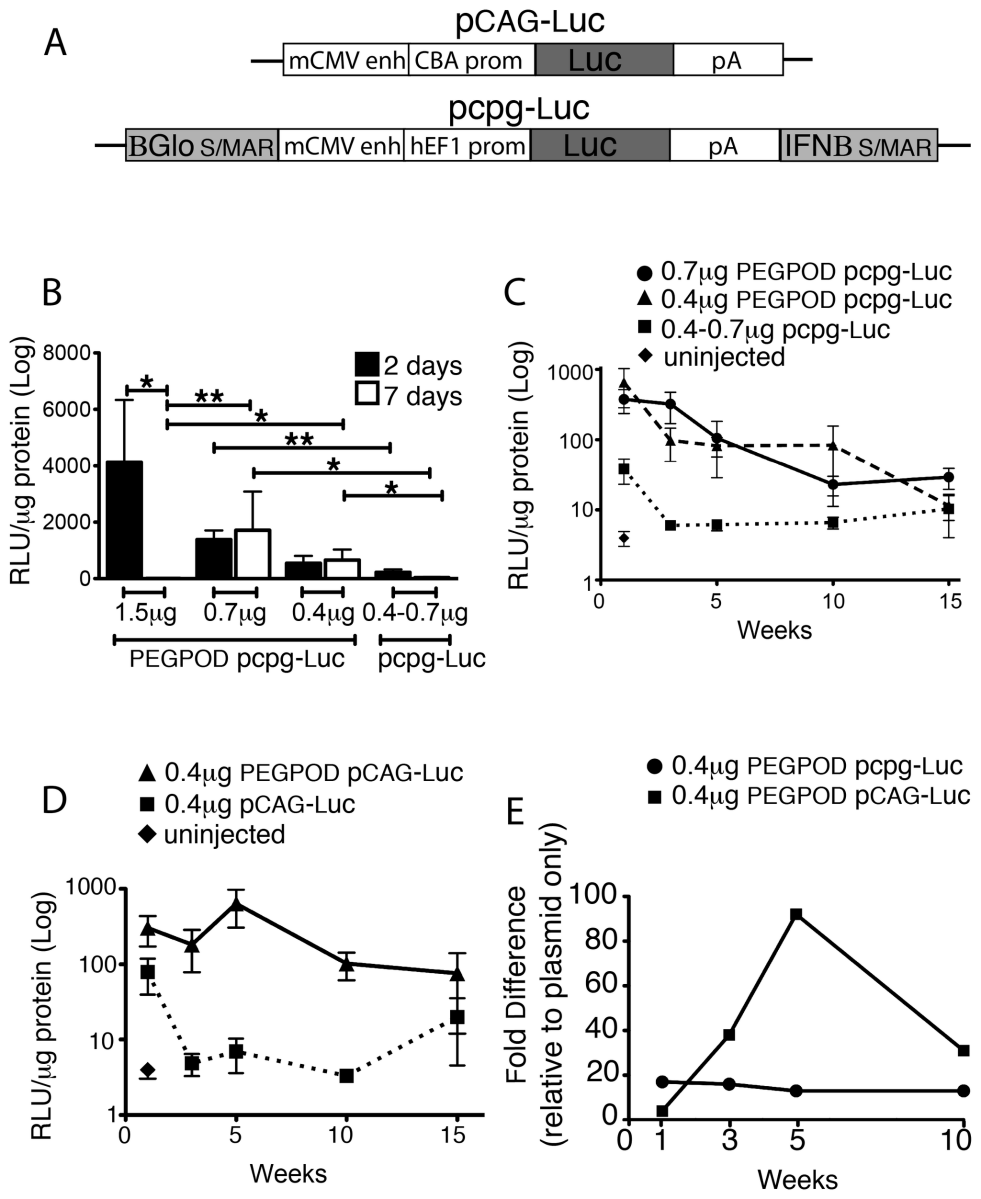
Transgene expression from PEGPOD DNA nanoparticles is dose-dependent and not influenced by S/MARs and CpG depletion. (A) Schematic of pCAG-Luc and pcpg-Luc. (B) Luciferase expression quantified at 48 hours and 7days post injection at three different concentrations (1.5, 0.7, and 0.4µg) of PEGPOD pcpg-Luc NPs into the murine subretinal space. Luciferase activity for non-compacted plasmid, pcpg-Luc, is also shown. While expression from PEGPOD pcpg-Luc NPs containing 1.5μg plasmid at 7days post-injection was significantly reduced relative to that at 48 hours (p<0.01), PEGPOD pcpg-Luc NPs containing either 0.7 or 0.4μg of plasmid did not show a reduction in luciferase expression at 7 days relative to 48 hours. In addition, expression from PEGPOD pcpg-Luc NPs containing 0.7 and 0.4μg of plasmid at 7 days is significantly higher (p<0.005 and p<0.05, respectively) than expression at 7 days from injections with PEGPOD pcpg-Luc NPs containing 1.5μg plasmid. 1.5μg, n=4; 0.7μg, n=9; 0.4μg, n=7. Data is shown as mean +/-SEM. (C) Quantification of luciferase expression at different time points up to 15 weeks post-injection at 0.7 and 0.4μg of PEGPOD pcpg-Luc NPs or non-compacted pcpg-Luc into the murine subretinal space. Injection of 0.4μg and 0.7µg PEGPOD pcpg-Luc NPs achieves statistically significant gene expression relative to non-compacted pcpg-Luc at each time point up to 10 weeks (p<0.05 for all time points). Average n per time point: 0.7μg, n=8; 0.4μg, n= 6; non-compacted plasmid, n=10; uninjected n=6. Data is shown as mean +/-SEM. (D) Quantification of luciferase at different time points up to 15 weeks post-injection of 0.4μg of PEGPOD pCAG-Luc NPs or non-compacted pCAG-Luc into the murine subretinal space. Injection of 0.4μg PEGPOD pCAG-Luc achieves significantly increased gene expression relative to injection with pCAG-Luc alone up to 10 weeks (p<0.05). Average n per time point: 0.4μg, n= 6; non-compacted plasmid, n=4; uninjected n=6. Data is shown as mean +/-SEM. (E) Graph of fold-difference in luciferase expression in retinas injected with 0.4µg of PEGPOD pcpg-Luc NPs and 0.4µg PEGPOD pCAG-Luc NPs relative to non-compacted plasmid at 1, 3, 5, and 10 weeks post-injection. S/MAR, scaffold/matrix attachment region; BGlo, ßglobin; mCMV enh, murine cytomegalovirus enhancer; pAn, polyAdenylation signal; IFNB, interferon ß; RLU, relative luciferase units.

Previously, we had described the use of PEGPOD DNA nanoparticles (NPs) constructed using pCAG-Luc (Figure 1A), a plasmid expressing luciferase from the CMV enhancer/ chicken β actin promoter for non viral gene transfer to the retina [19]. Such PEGPOD pCAG-Luc NPs containing 1.2μg DNA achieved maximum transgene expression at 48 hours post injection *in vivo*. At 7 days post injection, there was minimal transgene expression [19]. Scaffolid/ matrix attachment regions (S/MARs), originally used to retain episomal non viral vectors for stable gene expression *in vitro* [21], have shown efficacy in maintaining stable gene expression in a variety of cell types *in vivo* (reviewed in 22). A reduction in the CpG dinucleotide content of bacterial sequences in plasmid DNA has been shown to significantly reduce the host inflammatory response [23]. In recent studies, pcpg-MCS, a plasmid containing a S/MAR derived from the 5’ end of the human ß-globin gene (ßGlobin) and a S/MAR derived from the 5’ end of the human β-interferon gene (IFNβ) on a CpG-depleted backbone enables extended duration of transgene expression in rat cornea, liver and pancreatic ß cells [24–26]. In order to investigate whether similar strategies may extend the duration of transgene expression from PEGPOD DNA NPs, we utilized an identical plasmid expressing luciferase from a human Elongation Factor 1 (hEF1) promoter on a CpG-depleted backbone containing a ßGlobin S/MAR and an IFNß S/MAR (pcpg-Luc, Figure 1A). PEGPOD pcpg-Luc NPs were injected into the subretinal space of adult (6-8 weeks old) Balb/C mice at three different concentrations, specifically 1.5μg, 0.7μg or 0.4 μg DNA. Eyes were harvested at either 48 hours or 7 days post injection and total luciferase activity was quantified. We found that at concentrations of DNA similar to that used in our previous studies, ie. 1.5μg, there was no significant transgene expression at 7 days (4.6 RLU/μg) relative to that at 48 hours (4124.0 RLU/μg, p<0.01) from PEGPOD pcpg-Luc NPs, ie. the use of S/MARs and CpG depletion failed to extend transgene expression beyond that observed in our previous study using PEGPOD pCAG-Luc NPs. We did note however, that at lower concentrations of DNA i.e. 0.7μg or 0.4 μg, transgene expression from PEGPOD pcpg-Luc NPs at 7 days was not significantly less than that observed at 48 hours, i.e. 1716 and 1381 RLU/μg at 7days and 48 hours respectively for 0.7μg DNA or 565.7 and 543.4 RLU/μg at 7days and 48 hours respectively for 0.4μg DNA (Figure 1B). Furthermore, the levels of transgene expression observed at the lower concentrations of DNA at 7 days exceeded that seen using 1.5μg of DNA respectively (p <0.005 at 0.7μg and p<0.05 at 0.4μg). These data suggest that while smaller amounts of total DNA lead to less luciferase expression, the duration of such expression is extended.

### Long term transgene expression from PEGPOD DNA nanoparticles

Given that the duration of transgene expression from PEGPOD pcpg-Luc NPs may be concentration dependent, we next examined transgene expression from PEGPOD pcpg-Luc NPs containing 0.7μg or 0.4 μg DNA over 15 weeks. Specifically, we injected PEGPOD pcpg-Luc NPs into the subretinal space of adult (6-8 weeks old) Balb/C mice and harvested eyes for quantification of total luciferase at 1, 3, 5, 10, and 15 weeks post-injection (Figure 1C). As negative controls, we injected non-compacted pcpg-Luc plasmid DNA. We found that luciferase expression from PEGPOD pcpg-Luc NPs at both 0.7μg or 0.4 μg DNA was significantly (p<0.05 at all time points) higher for up to 10 weeks post injection relative to non-compacted pcpg-Luc plasmid DNA. Whereas luciferase expression from PEGPOD pcpg-Luc NPs containing 0.7μg DNA was 3.5 fold above that from non-compacted pcpg-Luc plasmid DNA at 10 weeks, luciferase expression from PEGPOD pcpg-Luc NPs containing 0.4μg DNA was 12.8 fold relative to that from non-compacted pcpg-Luc plasmid DNA. These data corroborate that the concentration of DNA compacted in PEGPOD pcpg-Luc NPs significantly influences duration of transgene expression *in vivo*.

### The plasmid backbone does not significantly influence duration of transgene expression from PEGPOD DNA nanoparticles

The above studies were initiated in order to explore the use of S/MARs and CpG-depletion on the duration of transgene expression from PEGPOD DNA NPs. However, given that our data suggested that there may be a more significant influence from DNA concentration relative to the presence or absence of SMARs or CpGs, we were motivated to perform studies examining luciferase expression from PEGPOD pCAG-Luc (a plasmid not modified to contain S/MARs or deleted for CpGs) NPs containing lower concentrations of DNA. Specifically, we injected PEGPOD pCAG-Luc NPs containing 0.4 μg DNA into the subretinal space of adult (6-8 weeks old) Balb/C mice and harvested eyes for quantification of total luciferase at 1, 3, 5, 10, and 15 weeks post-injection (Figure 1D). As negative controls, we injected 0.4μg non-compacted pCAG-Luc plasmid DNA. We found that expression from PEGPOD pCAG-Luc NPs containing 0.4μg DNA was 31 fold above that of non-compacted pCAG-Luc plasmid DNA at 10 weeks (p<0.05). The total levels of luciferase expression from PEGPOD pCAG-Luc NPs was not significantly different to luciferase expression from PEGPOD pcpg-Luc NPs at 10 weeks, suggesting that the S/MARs or deletion of CpGs were playing no significant role in extending duration of transgene expression from 48 hours to 10 weeks from PEGPOD DNA NPs. In contrast, our data strongly suggest that concentration of DNA in the PEGPOD DNA NPs is a more critical parameter and that there is an inverse correlation between concentration of DNA and longevity of transgene expression.

### The plasmid backbone influences stability of transgene expression from PEGPOD DNA nanoparticles

Although we found that S/MARs combined with CpG depletion did not contribute towards extending duration of transgene expression from PEGPOD pcpg-Luc NPs, we did find that such modifications of the plasmid backbone influenced *stability* of transgene expression. When we compared relative levels of luciferase expression from PEGPOD DNA NPs with their respective non-compacted plasmid DNA controls, we found that whereas there was a consistent relative difference for PEGPOD pcpg-Luc NPs over the 10 week period, relative levels of transgene expression from PEGPOD pCAG-Luc NPs fluctuated over this same time, with a peak at approximately 5 weeks (Figure 1E). No such relative peak of gene expression was observed from PEGPOD pcpg-Luc NPs (Figure 1E). 

We conclude that the final concentration of the total DNA compacted in PEGPOD DNA NPs is a potentially more important parameter than the use of S/MARs or CpG depletion in extending transgene expression from PEGPOD DNA NPs. However, since PEGPOD DNA NPs containing S/MARs and/or CpG depletion lead to more stable transgene expression (and additional reasons discussed below), we elected to continue our studies using PEGPOD pcpg-Luc NPs. 

### Localization of transgene expression from PEGPOD pcpg-Luc nanoparticles

**Figure 2:**
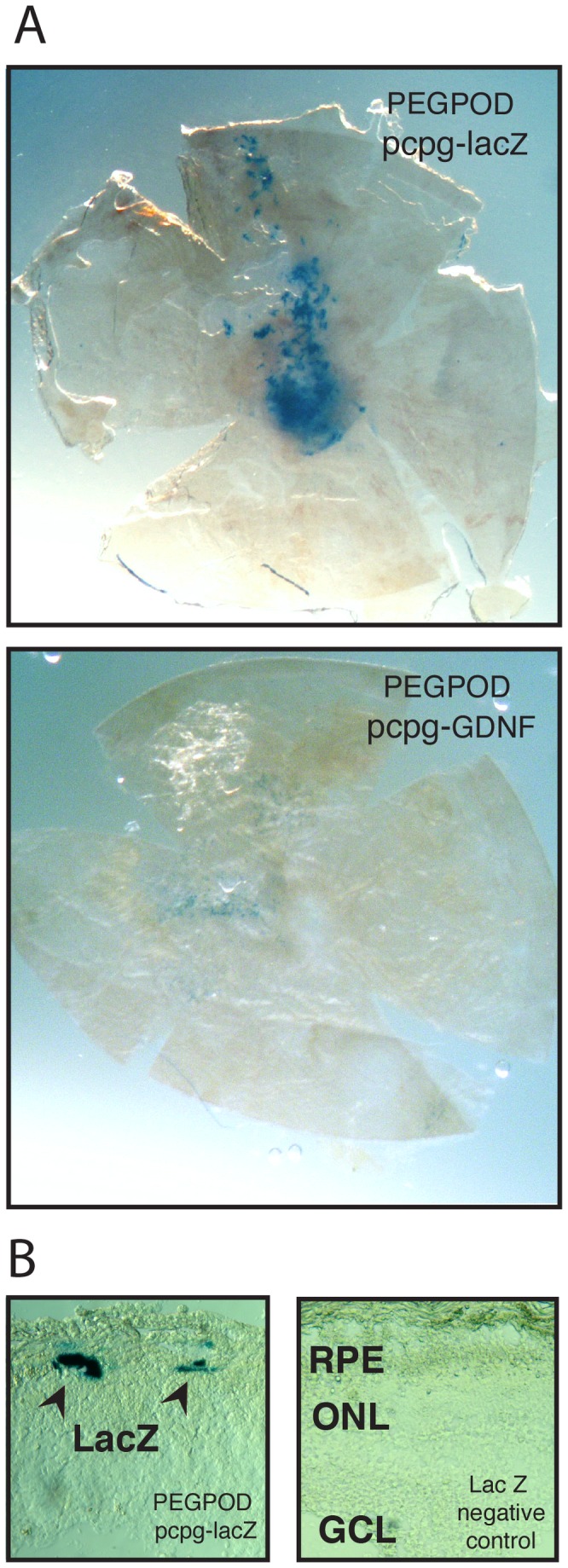
Localization of transgene expression from the human EF1 promoter in PEGPOD pcpg-LacZ. (A) Flatmounts of RPE/Choroid/Sclera from Balb/C mice injected in the subretinal space with PEGPOD pcpg-LacZ or PEGPOD pcpg-GDNF NPs assayed for LacZ activity 48 hours post injection. PEGPOD pcpg-LacZ injected eyes, but not PEGPOD pcpg-GDNF injected eyes, show strong LacZ activity throughout the eyecup. (B) Transverse sections through the retina of eyes injected with PEGPOD pcpg-LacZ show LacZ expression along the RPE with no expression in the retina. No LacZ activity was observed in retinal sections of eyes injected with control NPs. RPE, retinal pigment epithelium; ONL, outer nuclear layer; GCL, ganglion cell layer.

Previously, we have found that transgene expression from the chicken β actin promoter (CAG) in the context of PEGPOD pCAG-Luc NPs occurs primarily in the RPE [19]. In contrast, we have found that the CAG promoter enables transgene expression in the photoreceptors as well as the RPE when the transgene is delivered via a viral vector [27]. This data suggested that PEGPOD DNA NPs exclusively transduce RPE cells following delivery into the subretinal space of mice. As such, in the context of photoreceptor degeneration, PEGPOD DNA NPs are currently limited to the use of genes whose product may be secreted from the RPE. One class of such gene products includes growth factors such as glial cell line-derived neurotrophic factor (GDNF). In order to examine the location of gene expression from the human Elongation Factor 1 (hEF1) promoter in the context of PEGPOD DNA NPs, we replaced the luciferase cDNA in pcpg-Luc with that of LacZ or rat GDNF, generating PEGPOD pcpg-LacZ and PEGPOD pcpg-GDNF NPs respectively. PEGPOD pcpg-LacZ or PEGPOD pcpg-GDNF NPs were injected into the subretinal space of adult Balb/C mice and 48 hours later, eyecups were harvested and assayed for LacZ activity. Flatmounts of the eyecup with the retina removed indicated widespread LacZ expression from PEGPOD pcpg-LacZ injected eyes and as expected, no significant LacZ activity was detected in eyes injected with PEGPOD pcpg-GDNF NPs. Cross sections of similarly-injected eyecups in which the retina was retained indicated that LacZ expression was limited to the RPE cells. We conclude that in the context of PEGPOD DNA NPs, the hEF1 promoter enables transgene expression exclusively from the RPE (Figure 2). 

### PEGPOD pcpg-GDNF nanoparticles attenuate Blue Light Induced Photoreceptor Degeneration at 3 weeks post injection

**Figure 3:**
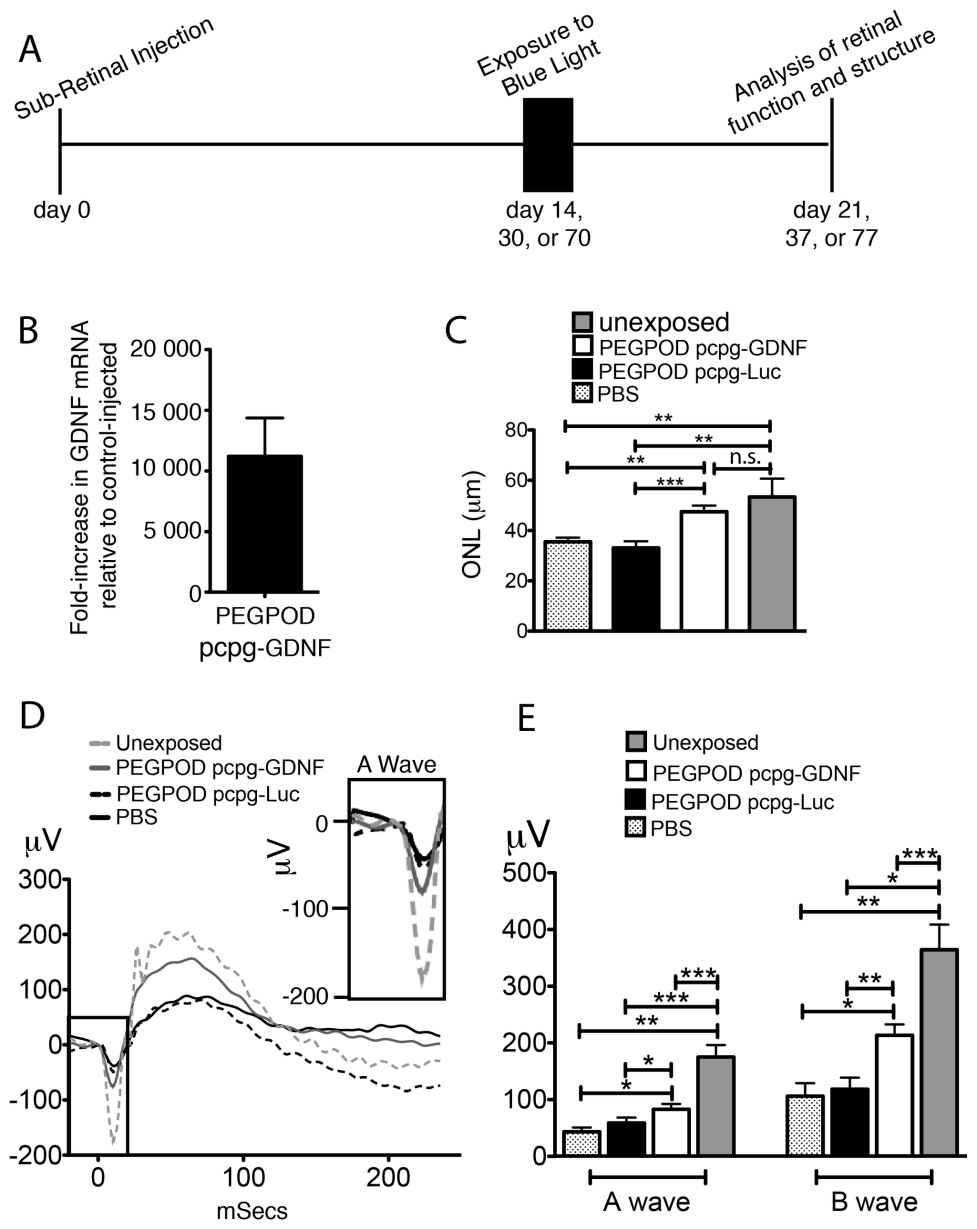
Injection of PEGPOD pcpg-GDNF NPs 14 days prior to blue light exposure provides complete structural and significant functional rescue of murine retina. (A) Schema depicting the time course of experiments described in this study (B) QRT-PCR analysis of GDNF mRNA at 14 days post injection of eyes with PEGPOD pcpg-GDNF NPs shows an 11,200 fold increase in GDNF signal relative to eyes injected with PBS. n=4 (C) Average ONL thickness of the superior hemisphere within 500μm of the optic nerve head 7 days following exposure to blue light. ONL thickness, measured at 21 days post injection, shows a significant increase in ONL thickness of retinas injected with PEGPOD pcpg-GDNF NPs relative to PBS and PEGPOD pcpg-Luc NP injected (p<0.01, p<0.0005, respectively). Unexposed eyes were injected with PBS 21 days prior to analysis and unexposed to blue light. PBS, n=5; PEGPOD pcpg-GDNF, n=13; PEGPOD pcpg-Luc, n=10. Data is shown as mean+SEM. (D) Average Erg tracings of eyes injected with PEGPOD pcpg-GDNF NPs, PEGPOD pcpg-Luc NPS, or PBS 14 days prior to light exposure were recorded at 7 days following exposure to blue light (21 days post injection). An amplified image of the boxed region of the ERG (A-wave) is also shown. Unexposed eyes were injected 21 days prior to analysis but unexposed to blue light. (E) Average A- and B-wave amplitudes of eyes injected with PEGPOD pcpg-GDNF NPs, PEGPOD pcpg-Luc NPs, or PBS 14 days prior to light exposure. PEGPOD pcpg-GDNF NP injected eyes show significantly increased A- and B-wave amplitudes relative to both PEGPOD pcpg-Luc NP injected (p<0.05, p<0.01 for A-wave and B-wave amplitudes, respectively) and PBS injected (p<0.05 for both A- and B-wave amplitudes) eyes. Data is shown as mean+SEM. ONL, outer nuclear layer; n.s., not significant.

Retinal diseases such as RP are genetically very heterogeneous. However, apoptosis is a well-documented common mechanism of photoreceptor cell death. Hence, there has been significant interest in the use of neurotrophic factors to attenuate apoptosis in RP [28]. One such commonly used factor is GDNF. However, high levels of GDNF can be toxic [29] and hence expression of low steady levels of GDNF may be preferred over variable or high levels of GDNF in a diseased retina. As described above, low and steady levels of transgene expression is a condition satisfied by PEGPOD DNA NPs that contain S/MARs on a CpG depleted plasmid backbone. We have previously described a murine model of blue light-induced photoreceptor degeneration that proceeds through caspase 3/7 mediated apoptosis [20]. Due to a limited duration of transgene expression, in our previous studies we injected PEGPOD DNA NPs 4 days prior to exposure of mice to blue light and measured photoreceptor degeneration at 1 week post light exposure [20]. In the current study we wished to determine whether the 4 day period post injection may be extended to 14, 30 or 70 days (Figure 3A).

Adult (6-8 week old) Balb/C mice were injected with either PEGPOD pcpg-GDNF NPs containing an average of 0.4μg DNA or PBS into the subretinal space. At two weeks post injection, mice were sacrificed and quantitative reverse transcription PCR (QRT-PCR) performed using primers designed to amplify rat GDNF cDNA. We found that the QRT-PCR signal in eyes injected with PEGPOD pcpg-GDNF NPs was 11,200-fold greater than that observed in PBS injected eyes (Figure 3B). 

Previously, we and others have documented that the injection procedure alone or injection of PBS is sufficient to confer some minor protection against photoreceptor degeneration in mice [20,30,31]. This is hypothesized to be caused by the release of growth factors following injury by the injection procedure [31]. Furthermore, we have also documented that PEGPOD DNA NPs expressing luciferase (PEGPOD pCAG-Luc) are also capable of conferring some protection against blue light induced apoptosis at a level beyond that achieved by PBS alone [20]. In order to account for these prior observations, in the current study we injected adult (6 week old) Balb/C mice with either PBS, PEGPOD pcpg-Luc NPs or PEGPOD pcpg-GDNF NPs. A total of 14 days later, we exposed mice to blue light and measured potential functional and histological rescue of the retina at 7 days post light exposure (21 days post injection). Specifically, following measurement of the scotopic electroretinogram (ERG), eyes were harvested, cryosectioned, and stained with DAPI for measurement of the thickness of the outer nuclear layer (ONL), a surrogate marker for the number of photoreceptor cells remaining in the retina.

In blue-light induced retinal degeneration, the majority of apoptosis and subsequent photoreceptor cell death occurs in the superior retina proximal to the optic nerve head [32]. Hence, we measured the average thickness of the ONL at 125μm, 250μm, and 500μm from the optic nerve head within the superior hemisphere (Figure 3C). We found that PEGPOD pcpg-GDNF NP injected eyes had 34% greater ONL thickness relative to PBS (p<0.01) and 44% greater ONL thickness relative to PEGPOD pcpg-Luc NP injected eyes (p<0.0005). Of significance also was the observation that there was no significant difference (Figure 3C) between the ONL thickness of PEGPOD pcpg-GDNF NP injected eyes and eyes not exposed to blue light, i.e. PEGPOD pcpg-GDNF conferred complete protection from blue light induced apoptosis 

Functional analysis of retinas by scotopic ERGs performed at 7 days post light exposure (day 21 post injection) revealed that PEGPOD pcpg-GDNF NP injected eyes had 92% (p<0.05) greater A-wave and 102% (p<0.05) greater B-wave amplitudes relative to PBS injected eyes and 41% (p<0.05) greater A-wave and 80% (p<0.01) greater B-wave amplitudes relative to PEGPOD pcpg-Luc NP injected eyes (Figure 3D, E). Although PEGPOD pcpg-GDNF mediated rescue was complete in terms of measurement of the ONL thickness, this level of histological rescue did not translate to complete rescue of the ERG. PEGPOD pcpg-GDNF injected eyes had an A and B wave amplitude of 52% and 41% respectively (p<0.0001 for both) to that of eyes not exposed to blue light. We conclude that delivery of PEGPOD pcpg-GDNF NPs 2 weeks prior to blue light exposure protects photoreceptor cells almost completely from degeneration when measured by histology and provides significant functional protection as determined by scotopic ERGs. These data significantly extend our previous observations of PEGPOD DNA mediated attenuation of photoreceptor degeneration. 

### Structural and functional rescue is sustained in PEGPOD pcpg-GDNF injected animals at 37 days post injection

**Figure 4:**
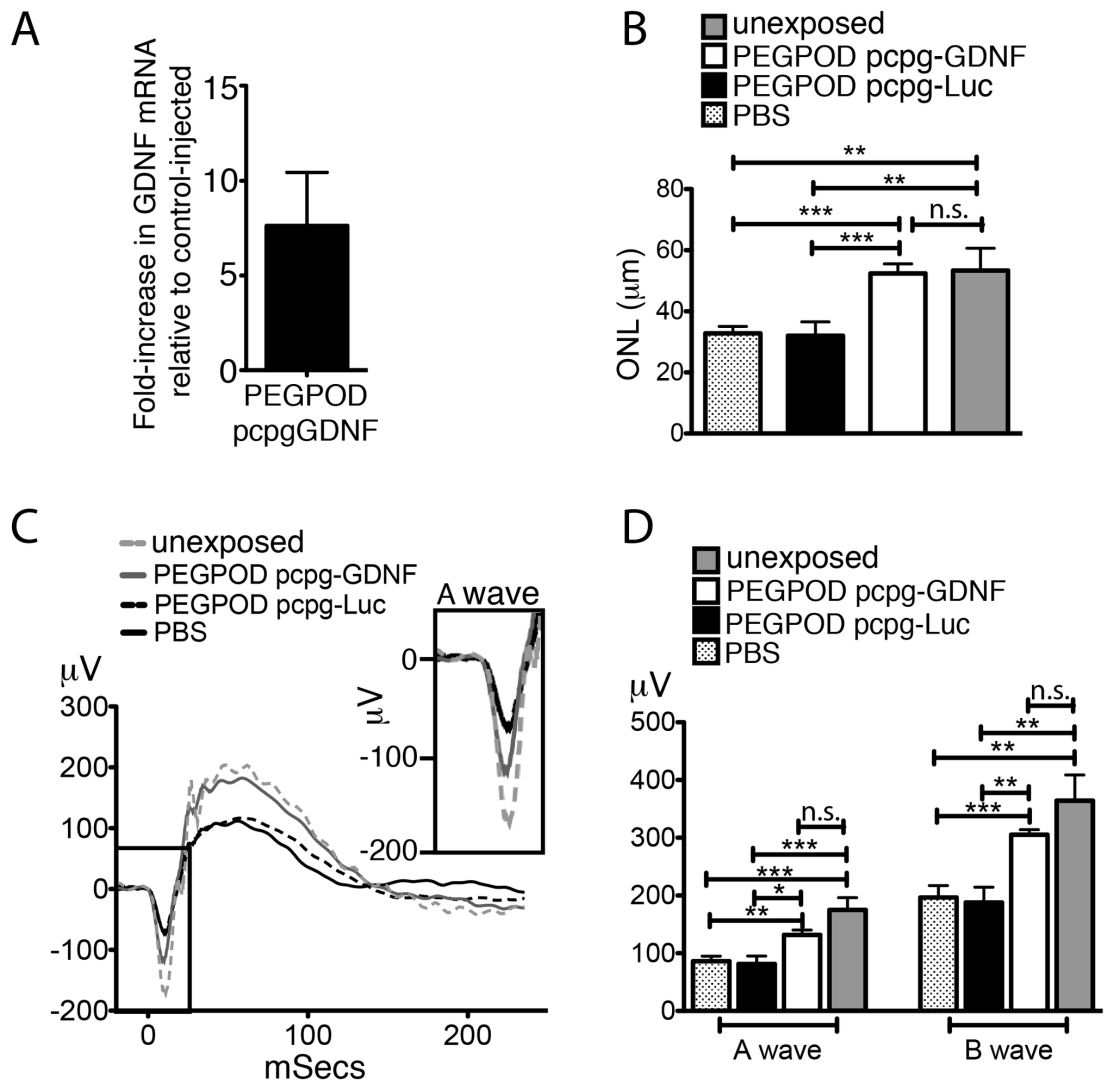
Complete histological and functional rescue following blue light exposure is observed at 37 days post injection of PEGPOD pcpg-GDNF NPs. (A) QRT-PCR analysis of GDNF mRNA in PEGPOD pcpg-GDNF NP injected eyes at 30 days post injection shows a 7.6 fold higher GDNF signal than that from eyes injected with PBS. n=4. (B) Average ONL thickness of the superior hemisphere within 500μm of the optic nerve head is significantly increased in eyes injected with PEGPOD pcpg-GDNF NPs at 30 days prior to light exposure relative to PBS injected eyes (p<0.0001) and PEGPOD pcpg-Luc NP injected eyes (p<0.0001). ONL thickness was measured at 7 days post light exposure. Unexposed eyes were not exposed to light, but were injected with PBS. PBS, n=9; PEGPOD pcpgGDNF, n=12; PEGPOD pcpg-Luc, n=10. Data is shown as mean+SEM. (C) Average ERG tracings of eyes injected with PEGPOD pcpg-GDNF NPs, PEGPOD pcpg-Luc NPS, or PBS and exposed to light 30 days after injection are shown. ERGs were performed 7 days following light exposure. (D) The average A- and B-wave amplitudes of eyes injected with PEGPOD pcpg-GDNF NPs are significantly increased relative to those of PBS injected eyes (p<0.005, p<0.0005 for A- and B-waves respectively) and PEGPOD pcpg-Luc NP injected eyes (p<0.01, p<0.005 for A- and B-waves, respectively). Unexposed eyes were injected with PBS. Data is shown as mean+SEM. ONL, outer nuclear layer; n.s., not significant.

QRT-PCR analyses of GDNF mRNA at 30 days post injection of PEGPOD pcpg-GDNF NPs into Balb/C mice showed a 7.6 fold higher level of GDNF signal relative to PBS injected mice (Figure 4A). Although GDNF expression was substantially lower at 30 days post injection relative to 14 days, we wished to examine whether these lower levels of expression were sufficient to protect mice from blue light induced photoreceptor degeneration. Balb/C mice were exposed to blue light at 30 days post injection and ocular tissues harvested 7 days later, immediately after measurement of the scotopic ERG. The average thickness of the ONL at 125μm, 250μm, and 500μm from the optic nerve head within the superior hemisphere of PEGPOD pcpg-GDNF NP injected eyes was 60% (p<0.0001) and 64% (p<0.0001) greater relative to that of PBS and PEGPOD pcpg-Luc NP injected mice, respectively (Figure 4B). We noted that whereas PBS or PEGPOD pcpg-Luc NP injected mice exposed to blue light exhibited a significant (p<0.004) loss of ONL thickness relative to mice not exposed to blue light, there was no significant loss of ONL in PEGPOD pcpg-GDNF NP injected eyes relative to eyes not exposed to blue light, i.e. there was complete rescue of the ONL in PEGPOD pcpg-GDNF NP injected eyes following exposure to blue light.

Measurement of the scotopic ERGs at 37 days post injection indicated that PEGPOD pcpg-GDNF NP injected eyes had 53% (p<0.005) greater A-wave and 55% (p<0.0005) greater B-wave amplitudes relative to PBS injected eyes and 62% (p<0.01) greater A-wave and 62% (p<0.005) greater B-wave amplitudes relative to PEGPOD pcpg-Luc NP injected eyes (Figure 4C, D). In addition, at 37 days post injection of PEGPOD pcpg-GDNF NPs there was no significant difference in either A- or B-wave amplitudes relative to unexposed eyes, indicating a complete functional rescue at this time point. 

### Structural but not functional rescue is sustained in PEGPOD pcpg-GDNF NP injected animals at 77 days post injection

**Figure 5:**
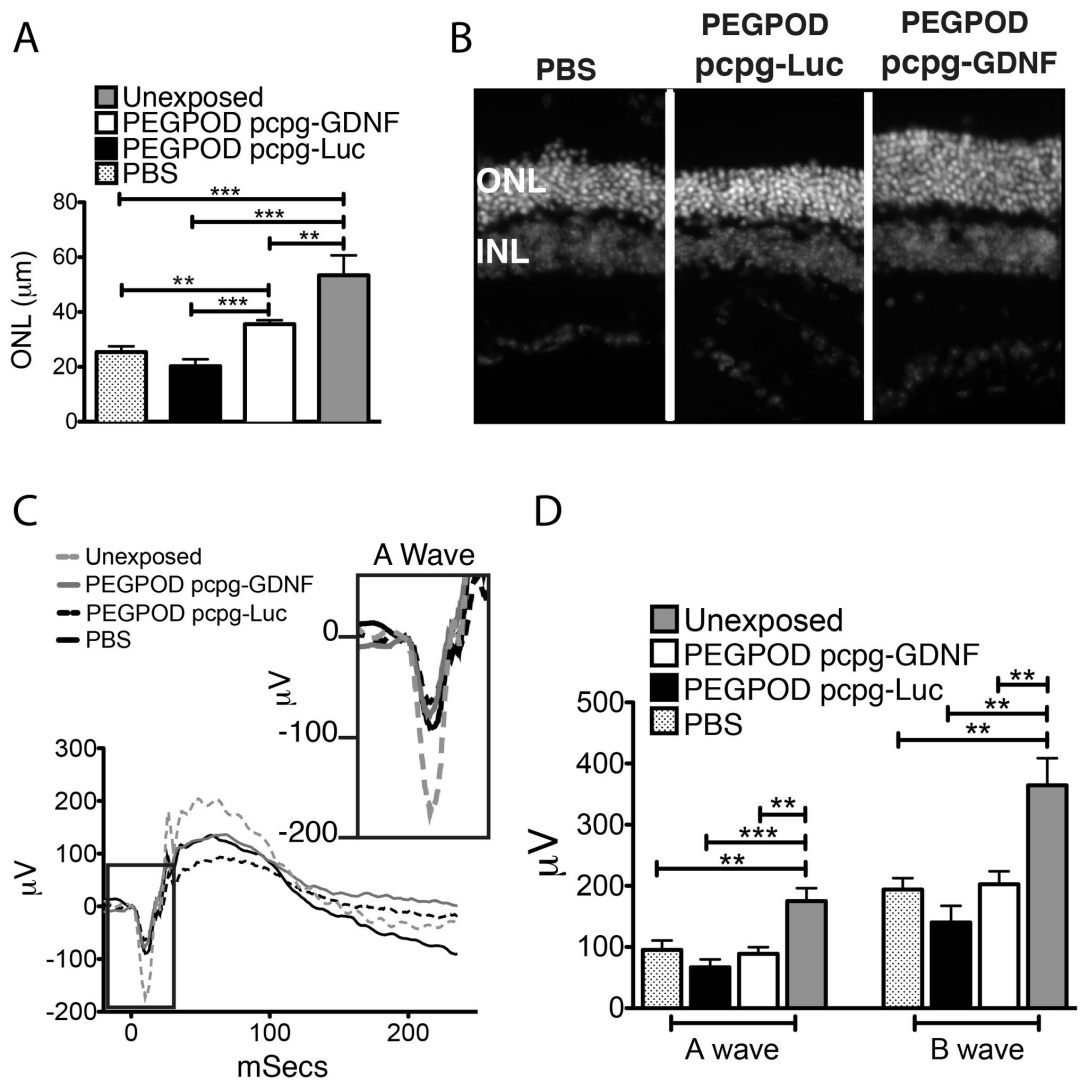
Histological, but not functional, rescue is observed in eyes injected with PEGPOD pcpg-GDNF 70 days prior to light exposure. (A) Average ONL thickness measured within 500μm of the optic nerve head of the superior hemisphere of the retina at 7 days post light exposure shows a significant increase in ONL thickness in eyes injected with PEGPOD pcpg-GDNF NPs relative to PBS (p<0.001) and PEGPOD pcpg-Luc NP injected (p<0.0001). Data is shown as mean+SEM. Unexposed eyes were injected with PBS. PBS, n=7; PEGPOD pcpg-GDNF, n=15; PEGPOD pcpg-Luc, n=9. (B) Representative retinal sections of eyes injected with PEGPOD pcpg-GDNF NPs, PEGPOD pcpg-Luc NPs, or PBS 70 days prior to light exposure and stained with DAPI are shown. (C) Average ERG tracings of eyes injected with PEGPOD pcpg-GDNF NPs, PEGPOD pcpg-Luc NPs, or PBS and exposed to light 70 days post injection are shown. ERGs were performed 7 days post light exposure. (D) Average A- and B-wave amplitudes of ERGs recorded from eyes injected with PEGPOD pcpg-GDNF NPs, PEGPOD pcpg-Luc NPs, or PBS and exposed to light 70 days post injection are shown. There is no significant difference between A- and B-wave amplitudes in the PEGPOD pcpg-GDNF NP injected eyes relative to PBS injected or PEGPOD pcpg-Luc NP injected eyes. Unexposed eyes were injected with PBS. Data is shown as mean+SEM. ONL/INL, outer/inner nuclear layer.

At 70 days post injection, QRT-PCR analysis of GDNF mRNA from PEGPOD pcpg-GDNF NP injected Balb/C mice indicated no significant difference in GDNF signal relative to PBS injected mice (data not shown). Nonetheless, we proceeded to examine any potential rescue of PEGPOD pcpg-GDNF NP injected mice using the same criteria as those described above. At 7 days post light exposure (77 days post injection), the average thickness of the ONL at 125μm, 250μm, and 500μm from the optic nerve head within the superior hemisphere of PEGPOD pcpg-GDNF NP injected eyes was 40% (p<0.001) and 76% (p<0.0001) greater relative to PBS or PEGPOD pcpg-Luc NP injected mice respectively (Figure 5A, B). This result suggests that the levels of GDNF necessary for protection of the ONL are below the detection limits of our QRT-PCR, which may be explained given that the RT-PCR signal is being derived from the entire eye whereas the thickness of the ONL is being reported from the region undergoing greatest apoptosis in the blue light induced model of retinal degeneration. We noted that while at 21 days and 37 days post-injection, there was complete anatomical (ONL thickness) rescue in PEGPOD pcpg-GDNF NP injected eyes relative to eyes not exposed to blue light, at 70 days post-injection, the ONL thickness of PEGPOD pcpg-GDNF NP injected eyes was 77% (p<0.001) that of eyes not exposed to blue light. 

In contrast to the significant rescue of the ONL, we found no significant differences in either the A- or B-wave amplitudes between PEGPOD pcpg-GDNF NP injected and PBS or PEGPOD pcpg-Luc NP injected mice respectively (Figure 5C, D). We conclude that the levels of GDNF expression in the region of injection are below the detection limits of our QRT-PCR assay from whole eyes but the levels in the region of injection are sufficient to confer histological rescue of blue light induced photoreceptor degeneration. We also conclude that full field ERGs are too spatially insensitive for detection of differences between PEGPOD pcpg-GDNF NP injected eyes and PBS or PEGPOD pcpg-Luc NP injected eyes at 77 days post injection. 

## Discussion

In this study, we report that expression of luciferase from PEGPOD DNA NPs can be extended from 48 hours to more than 10 weeks - a significant step towards the further development of PEGPOD NPs for gene transfer. When the luciferase cDNA was substituted with a transgene encoding GDNF, the level of transgene expression achieved was sufficient to enable histological rescue of blue light induced retinal degeneration at 77 days post injection. In these same studies, histological as well as electrophysiological (ERG) rescue could be measured at 37 days post injection. As discussed below in some detail, there is currently limited but encouraging evidence that non-viral vectors are efficacious in long-term rescue of photoreceptor degeneration and the studies presented in this report add significantly to the reported progress on the development of non-viral gene transfer for the retina.

To extend the duration of transgene expression from PEGPOD DNA NPs, we examined modifications to the plasmid backbone, i.e. inclusion of S/MARs as well as a reduction in its CpG content and titration of the concentration of DNA compacted into the NP. We were successful in achieving a 35 fold increase in duration of expression of luciferase. We found that the duration of transgene expression was inversely correlated with the concentration of the PEGPOD DNA NP and not significantly influenced by the presence of IFNß and ßglobin S/MARs on a CpG-depleted plasmid backbone. However, the presence of S/MARs and a reduction of CpG content did influence the stability of luciferase expression from the PEGPOD pcpg-Luc NP, which was maintained at ~ 13 fold above that of non-compacted pcpg-Luc up to 10 weeks post injection. 

Naash and colleagues have previously reported that injection of a pegylated poly lysine (CK30) NP into adult mice enables transgene expression in the RPE 3-fold above naked DNA for up to 30 days [33]. Introduction of the human IFNβ S/MAR into the plasmid compacted with CK30 NPs increased longevity of transgene expression in the RPE above the non-S/MAR containing plasmid for up to 360 days but there was no significant difference in transgene levels up to 120 days between CK30 NPs and naked DNA [34]. That study employed the pEPI plasmid backbone, one of the first systems to demonstrate the use of S/MARs for increased maintenance of gene expression [21]. Since its original inception, however, pEPI has undergone a number of modifications to enhance performance including a reduction in CpG content [35] and localization of S/MAR to its core element [36]. A number of recent studies have successfully employed a CpG-depleted plasmid (pcpg-MCS; the same as that employed in this study) containing a human IFNβ S/MAR, in addition to a human ßGlobin S/MAR [24–26]. 

The plasmid pcpg-MCS outperformed pEPI in duration of transgene expression when delivered to rat corneas *in vivo* [24]. However, in that study, the pEPI plasmid performed no better than its non-S/MAR counterpart, further evidence of the idiosyncratic nature of S/MAR function in different systems. Similar to our study, Yurek and colleagues [37] found no significant difference in either the levels or duration of luciferase expression from the pcpg-MCS plasmid when delivered to the mouse striatum using CK30 NPs relative to a non-S/MAR containing plasmid up to 11 weeks following injection, although stable luciferase expression up to 1 year post injection was reported for the S/MAR-containing plasmid. When the luciferase cDNA was replaced by a transgene encoding GDNF and injected via CK30 NPs into rat striatum, GDNF expression was sustained for 6 months [38]. As was the case in our study, the consistency observed in the level of luciferase expression was not observed for GDNF, i.e. GDNF expression fell sharply from an initial peak of approximately 16-fold above baseline at 7 days to approximately 6-fold above baseline at 14 days and continued to decline out to 180 days post-injection [38], indicating that the stability of transgene expression may also be dependent upon the specific protein being expressed. 

We did not observe any change in localization of transgene expression from the PEGPOD pcpg-LacZ NP from that reported by us previously [19] for PEGPOD pCAG-LacZ. Expression from PEGPOD pcpg-LacZ NP was exclusively in the RPE, indicating that localization of expression was also not influenced by the presence of S/MARs on a CpG-depleted backbone. Localization of gene expression in the photoreceptors is relevant in the context of treating RP, since the majority of genes involved in RP are photoreceptor-specific genes. Naash and colleagues have previously reported use of CK30 NPs for highly efficient gene transfer to the photoreceptors [39] of adult mice, rivaling or exceeding gene transfer levels achievable by viral vectors [40]. When CK30 NPs are injected into the subretinal space of P22 *rds* mice, these NPs enabled transgene expression levels approximately 2-fold above that of mice injected with non-compacted plasmid for up to 120 days post-injection [41]. This was sufficient to achieve modest functional and histological rescue. In a previous study, we found that pegylated NPs expressing luciferase generated with POD peptide enabled 215 fold higher levels of gene expression relative to naked DNA whereas Tat and CK30 NPs enabled 56 fold and 24 fold higher levels of luciferase expression relative to naked DNA subsequent to subretinal injection in adult mice [19]. Importantly, when the luciferase-expressing plasmid was exchanged with that of a marker gene (LacZ) to enable localization of transgene expression, we did not find any evidence of gene expression in the photoreceptor cells from either CK30, POD or Tat. Indeed, similar to the current study, we found evidence only of gene expression from the RPE [19] and in each case it was very limited relative to gene expression from recombinant viruses. In our hands, CK30 NPs transduced only the occasional RPE cell [19]. Other laboratories have also failed to observe transgene expression from photoreceptors using CK30 NPs (Wolfgang Baehr, personal communication). We have not been able to reconcile these different observations regarding CK30 NPs despite significant effort, including the use of reagents from the same source [39] as the original study (data not shown). 

Gene delivery to the RPE in order to rescue a photoreceptor disease is a compromise, but a potentially viable one when delivering a secreted neurotrophic factor such as GDNF. Considering the heterogeneity of etiological factors involved in retinal degeneration, in addition to the cost of developing gene-specific therapy, the benefit of delivering neuroprotective factors, such as GDNF, cannot be ignored. In addition, recent data from clinical gene therapy studies targeting Leber’s Congenital Amaurosis revealed continued degeneration despite treatment, suggestive of a potential benefit of supplementing gene-specific therapies with co-delivery of neuroprotective factors [42]. Delivery of ciliary neurotrophic factor (CNTF) to patients with advanced retinal degeneration via encapsulated intravitreal implants has been shown to be both safe and effective in phase I clinical trials [4], and is currently under clinical investigation also for geographic atrophy, an advanced form of macular degeneration [43], and achromatopsia (*clinical* *trials.gov*). Intravitreal delivery of recombinant CNTF has also recently been shown to significantly enhance the efficacy of gene augmentation therapy in the treatment of achromatopsia [44].

Gene delivery of neurotrophic factors to the retina, however, is not without its concerns. At higher doses, both CNTF and GDNF have been shown to be toxic [15,45]. At higher doses, CNTF has been shown to significantly reduce retinal function [15]. Both retinal thinning and reduction in ERG response has been observed from GDNF at higher doses [45]. GDNF has been shown to have a direct effect on the induction of tyrosine hydroxylase (TH) [46,47], an enzyme involved in the production of L-DOPA and expressed exclusively in RPE cells in the eye [48]. Through the production of nitrous oxide, L-DOPA has been shown to exhibit cytoxic effects on RPE cells *in vitro* [49], an effect more marked in amelanotic cells (such as occur in Balb/C mice) than in melanotic cells. Considering that the kinetics of GDNF expression differed from that of luciferase in both our study and that investigating CK30 NPs as a delivery vehicle for GDNF rescue of dopaminergic neurons in a model of Parkinson’s disease [37,38], it is reasonable to speculate that in general the duration of transgene expression would depend on the protein expressed and its effect at higher doses on the expressing cells. 

In summary, we have shown that luciferase expression from PEGPOD DNA NPs can be extended from 48 hours to more than 10 weeks and that PEGPOD DNA NPs expressing GDNF can enable histological rescue of blue light induced photoreceptor degeneration at 77 days post injection and histological and physiological level at 37 days post injection. Given the level of histological rescue observed at 77 days post injection, a more sensitive technique such as microelectrode array of isolated retina may enable a measurement of physiological rescue at this same time point. We have shown that the duration of transgene expression from PEGPOD DNA NPs is not significantly influenced by S/MARs or CpG depletion but that these elements do influence the stability of transgene expression. While the duration of transgene expression does not yet rival that achieved by viral vectors, these advancements are an important step forward for the use of PEGPOD DNA NPs and for the use of non-viral vectors in the retina. Our results may have future implications for the treatment of diseases such as RP or other diseases involving the degeneration of photoreceptor cells.

## Materials and Methods

### Animals

This study was carried out in strict accordance with the Statement for the Use of Animals in Ophthalmic and Vision Research, set out by the Association of Research in Vision and Ophthalmology (ARVO) and was approved by Tufts University Institutional Animal Care and Use Committee (IACUC) protocol B2011-150 and Tufts University Institutional Biosafety Committee registration 2011-BRIA68.

### Reagents

Peptide for Ocular Delivery (POD, CGGG(ARKKAAKA)_4_) was synthesized and HPLC purified by the Tufts University Peptide Synthesis Core Facility (Boston, MA). The plasmids pcpg-mcs and pcpg-LacZ were purchased from InvivoGen (San Diego, CA). Pcpg-GDNF was created by first inserting rat GDNF from pGDNF3a (graciously provided by M. Bohn, Children’s Memorial Hospital, Northwestern University, Chicago, IL) into pCDNA3.1 (Invitrogen, Carlsbad, CA) using KpnI/NotI. GDNF was then excised using Acc65I/XbaI and inserted into pcpg-mcs digested with Acc65I/NheI. pcpg-Luc was created by excising luciferase from pBluescript II KS (Stratagene, La Jolla, CA) (described in [19]) using Bsp1201/XbaI and inserting it into pcpg-mcs digested with Bsp1201/NheI. Methoxy-PEG-maleimide-10kD (PEG) was obtained from Nektar Transforming Therapeutics (San Carlos, CA). pCAG-Luc was created as described previously [19]. 

### Nanoparticle Construction

Plasmids were compacted with PEGPOD as described previously [19]. Briefly, pegylation of POD was performed by suspending the peptide in 0.1M sodium phosphate, pH 7.2, 5mM EDTA at 20mg/mL. An equimolar amount of PEG-maleimide resuspended in the same volume of DMSO was added drop-wise to the peptide, vortexing between drops. The mix was vortexed overnight and dialyzed into 0.1% TFA using a Biogel P6 column (BioRad). Peptide concentration was quantified using a BCA Protein Assay Kit (Thermo Scientific; Rockford, IL). Pegylated POD (PEGPOD) was used to compact DNA in a 1.8nmole:2μg ratio. Prior to compaction, the DNA was diluted in dH_2_O to a concentration of 0.18μg/mL and then added drop-wise with vortexing. The mix was dialyzed into 5% dextrose using Amicon Ultra 10K centrifugal filters (Millipore; Billerica, MA). To quantify the DNA in the compaction, nanoparticles were digested in 0.25% trypsin (Invitrogen) at 37°C for 15 minutes and then run on a 1% agarose gel. A plasmid standard curve was used to determine concentration. 

### PEGPOD-mediated delivery of DNA *in vivo*

Balb/C mice were purchased from Jackson Laboratories (Bar Harbor, ME), maintained in a 12 hour light-dark cycle unless otherwise stated, and cared for in accordance with federal, state, and local regulations. Male mice, 6-8 weeks old, were anesthetized by intraperitoneal injection of 100mg/Kg xylazine (Lloyd Inc.; Shenandoah, IA) and 1000mg/Kg ketamine (Ketaset, Fort Dodge Animal Health; Fort Dodge, IA) followed by topical application of Proparacaine (Akorn; Lake Forest, IL) to the cornea. Injections into subretinal space were performed with a 32G needle and a 5μl glass syringe (Hamilton; Reno, NV). 0.4-1.5µg DNA in nanoparticles compacted in a peptide:DNA ratio of 1.8nmole:2µg was injected in a volume of 2µl in 5% dextrose using a trans-scleral and trans-choroidal approach. 

### Characterization of PEGPOD delivered-DNA expression

To quantify luciferase expression, animals were injected with PEGPOD pcpg-Luc, pcpg-Luc PEGPOD pCAG-Luc, or pCAG-Luc. Animals were sacrificed 2 to 105 days after injection by CO_2_ inhalation followed by cervical dislocation. Eyes were harvested and dissected; the posterior eyecup was suspended in Homogenization Buffer (50mM Tris-HCl, pH 8.0, 150mM NaCl) with 20μL/mL protease inhibitors (Sigma; St. Louis, MO) and homogenized using a PowerMax AHS 200 homogenizer (VWR; West Chester, PA). Supernatants were assayed for luciferase activity as per manufacturer’s instructions (Promega; Madison, WI) using a Glomax 20/20 luminometer (Promega) over a 10 second integration. 

### mRNA quantification

For mRNA quantification, 0.4μg of PEGPOD pcpg-GDNF (in 2μL) or 2μL PBS were injected subretinally. 14, 30, or 70 days later, eyes were harvested and the posterior eyecup isolated. Eyes were homogenized as described above, and RNA was extracted using a Qiagen RNeasy Mini Kit (Qiagen, Valencia, CA). DNase treatment was performed using Turbo DNase (Ambion, Austin, TX) according to manufacturer’s instructions. Samples were prepared using an iscript One-Step RT-PCR kit and analyzed on an iQ-5 Thermal Cycler (Biorad, Hercules, CA). A rat GDNF primer/probe (Assay #Rn00569510_m1, Taq Man Gene Expression Assay, Applied Biosystems, Carlsbad, CA) and mouse GAPD FAM-MGB primer/probe (Applied Biosystems) were used. ∆Ct values were calculated (rat GDNF Ct or PBS Ct - GAPD Ct). ∆∆Ct values were calculated (∆Ct GDNF - ∆Ct PBS). To determine fold difference in GDNF mRNA expression, 2^-(∆∆ Ct)^ was calculated. 

### Light Degeneration Model

Mice were dark adapted for 4 days. Eyes were dilated with 1% Tropicamide (Akorn, Inc.). Mice were exposed to 450nm light (Bili Blue; Interelectric, Warren, PA) at an intensity of 4000-6000Lux (Light Meter, Lux/FC 840020; Sper Scientific, Scottsdale, AZ) for 90 minutes. 7 days later, mice were analyzed by electroretinography (ERG). After dark adaptation overnight, mice were anesthetized as described above. Eyes were dilated using 1% Tropicamide, and scotopic ERGs were recorded at 0dB using contact lens electrodes and the UTAS system with BigShot ganzfeld (LKC Technologies; Gaithersburg, MD). 5 flashes were averaged, and A- and B-wave amplitudes measured. For ONL thickness quantification, mice were sacrificed as above. Eyes were harvested, cauterized for orientation, and fixed in 4% paraformaldehyde. For sectioning, eyes were dehydrated with a sucrose gradient. Retinal sections were made by embedding tissues in Optimal Cutting Temperature Compound (Sakura Finetek; Torrance, CA) and sectioning at 14μm using a Microm 550 Cryostat along the vertical meridian. Nuclear counterstaining was performed with 4’6-diamidino-2-phenylindole (DAPI). Sections were imaged using an Olympus IX51 microscope with fluorescence filters and a Retiga 2000R FAST camera with QCapture Pro 5.0 (QImaging, British Columbia, Canada). ONL thickness was measured at 125, 250, and 500μm from the optic nerve head. Thickness was measured using Image J (National Institutes of Health; Bethesda, MD) software.

### Statistical analyses

All statistical analyses were performed using Prism Software 5.0a (GraphPad Software, Inc.; La Jolla, CA). Luciferase data proved to have significantly different variances as determined by an F-test. Therefore, a logarithmic transformation was performed to eliminate variability between the variances before a test of significance was performed. An ANOVA test followed by a Mann Whitney test was used for comparisons of more than 2 groups. For 2 groups, an unpaired t-test was used to determine significance. Due to the large variability between subretinal injections in mice, significant outliers were determined using Grubb’s Test and excluded (QuickCalc, GraphPad Software, San Diego, CA). For quantification of rescue, eyes exposed to blue light but exhibiting B-waves greater than the median B-wave of wild type mice (not exposed to blue light) were considered as not having degenerated as a result of light treatment, and were not included for quantification. ERG data was compared using an ANOVA test followed by Mann Whitney tests for significance. Average ONL thickness across the first 500µm of the superior hemisphere was calculated and compared using an ANOVA test followed by Mann Whitney tests for significance. The number of eyes per condition is reported (n).
